# Study of Ultrasonic Dispersion of Graphene Nanoplatelets

**DOI:** 10.3390/ma12111757

**Published:** 2019-05-30

**Authors:** Bin Zhang, Tijun Chen

**Affiliations:** State Key Laboratory of Advanced Processing and Recycling of Nonferrous Metals, Lanzhou University of Technology, Lanzhou 730050, China; zhangbin429@163.com

**Keywords:** graphene nanoplatelets, ultrasonic, dispersion, fragmentation, exfoliation

## Abstract

Graphene has outstanding mechanical properties due to its unique structure, and is regarded as an ideal reinforcement of metal matrix composites. However, it is always in an agglomerate form due to its large specific surface area, and thus, it must be first dispersed prior to combining with a matrix, and ultrasonic treatment is considered to be the most effective way. In this work, the effects of parameters of tip ultrasonic treatment, such as ultrasonic time, ultrasonic power, solvent kind, and its temperature, on dispersion and structure of graphene nanoplatelets (GNPs) were studied. The results show that increasing ultrasonic time or ultrasonic power can enhance the dispersion and exfoliation effects of GNPs, but also increase fragmentation degree and disorder degree of C-atom distribution simultaneously. Solvents with low temperature, low viscosity, or high surface tension have similar effects to those of increasing ultrasonic time or power. However, for tap water, a high-surface-tension solvent, it has relatively low fragmentation degree, and good dispersion and exfoliation effects due to the hydrophilicity of GNPs. However, ethyl alcohol is a more suitable solvent because it has excellent volatility and inert reaction characteristics with GNPs and matrix alloys besides a good dispersion effect. The GNPs can achieve the expected status when they are ultrasonically treated for 4 h under a power of 960 W in EA solvent at 35 °C.

## 1. Introduction

Graphene has excellent mechanical properties, such as ultra-high tensile strength of 125 GPa [1] and elastic modulus of 1 TPa, and thus it is considered to be an ideal reinforcement for metal matrix composites [2]. However, graphene is easily agglomerated due to its large specific surface area (2630 m^2^/g) and strong Van der Waal cohesive force [3], and is very difficult to disperse uniformly in a metal matrix [4]. Therefore, graphene agglomerates must be dispersed prior to combining with a matrix. A lot of efforts have been made to disperse the agglomerates. Huang et al. [5] used the in situ polymerization method to disperse graphene nanoplatelets (GNPs) by polarizing the layers of GNPs, which increases the gap between layers, thus preventing agglomeration. However, this method has strict requirements for solvents, and it is quite difficult to find suitable polymerization solvents. To overcome this difficulty, graphene functionalization has been conducted. One method is to graft some groups, such as carboxyl, hydroxyl, and epoxide groups, on the surface of graphene by covalency and non-covalency, i.e., leading the graphene to become graphene oxide, which has good hydrophilia property and can be easily uniformly dispersed in aqueous solutions [6,7,8,9,10]. Another method is to introduce a surfactant to form a thin film on the surface of graphene that separates them from each other by means of π–π stacking [11,12], i.e., these two methods need to add external dispersants, but the residual dispersants are difficult to remove even after being heat-treated or reduced. This must bring about a contamination, and thus impairs the mechanical properties of the resulting composite. Besides the above chemical dispersion methods, ultrasonic treatment has been proposed as promising. During ultrasonic treatment, a lot of cavitation bubbles are generated in the solution and then rapidly collapse, generating shockwaves that break down the agglomerates and separate the stacked graphene from each other [13,14]. In addition, ultrasonic treatment is convenient and, more importantly, is environmentally friendly, and it has no impurity to be introduced if a volatile solvent is used [15,16,17].

There are two types of ultrasonic equipment, tip and bath sonicator. The power of the tip sonicator is always higher than that of the bath one, and thus, the tip sonicator is much more efficient for dispersion than the bath sonicator under the same conditions [18]. However, most investigations emphasize the microstructure and mechanical properties of the achieved graphene reinforced composites [19,20,21]. As for the fabrication of the composites, especially for the dispersion of graphene, only a set of parameters was provided, and the detailed effects of the parameters such as the ultrasonic powder and time, the viscosity, surface tension and temperature of the solvents on the graphene dispersion, are still unclear. Therefore, the employed parameters in their investigation might not be the optimum, and the mechanical properties of the composites are unsatisfactory due to the resulting inhomogeneous distribution of graphene. Previous investigations indicated that ultrasonic treatment could disperse GNP agglomerates, but simultaneously lead them to fragmenting [22]. The fragmentation not only reduces the aspect ratio of graphene and decreases its load transfer efficiency, and thus, impairs its strengthening role [23], but also increases C-atoms with dangling bonds at the edge of GNPs; such C-atoms always have high chemical activity and can easily react with matrix-alloying elements to form brittle carbides at the graphene/matrix interface [24,25], which also impairs the strengthening role of GNPs [26]. In addition, some investigations have suggested that vacancies might form during ultrasonic treatment and the structure integrity of graphene was then destroyed, and thus the strengthening role was also decreased [27]. Cheng et al. found that the ultrasonic dispersion of carbon nanotubes was dependent on the solvent physical properties such as vapor pressure, viscosity, and surface tension [28]. Furthermore, solvent temperature rise is a common phenomenon during ultrasonic treatment, and the vapor pressure of a solvent has a close relationship with its temperature [29], i.e., the solvent temperature also can affect the dispersion of graphene. However, unfortunately, there are no investigations on these aspects.

Therefore, the effects of tip sonication parameters, such as ultrasonic power and time, solvent viscosity, surface tension, and temperature on the dispersion effect, especially on the structure of GNPs, were investigated in this work. The aim is to achieve a feasible technology for dispersing graphene or GNPs during fabrication of metal matrix composites.

## 2. Materials and Methods

**Figure 1:**
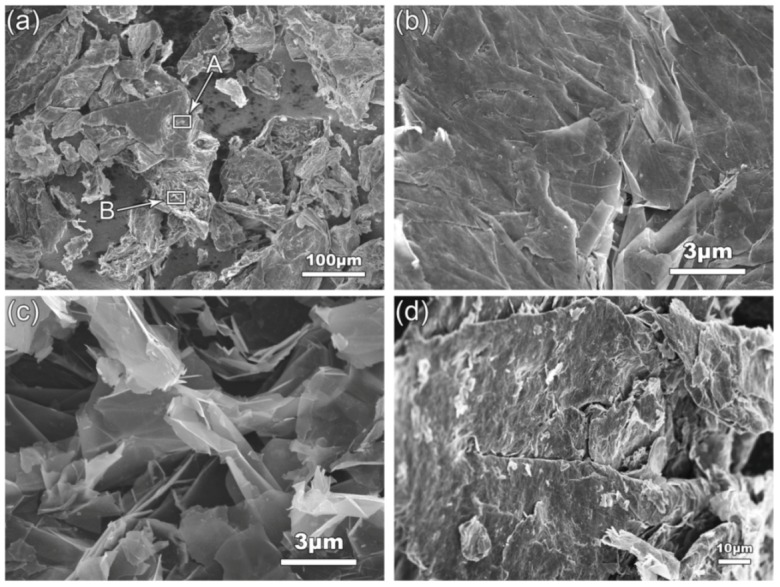
SEM images of the as-received GNPs. (**a**) the general morphology, (**b**) and (**c**) the high-magnification images corresponding to A and B in (**a**), and (**d**) graphite particle in the as-received GNPs.

The used GNPs were purchased from XFNANO technology, Nanjing, China and they have nominal sizes of 3–10 nm in thickness and 1–3 μm in diameter, and they are in a dry powder form. Figure 1 shows the scanning electron microscopy (SEM) images of the as-received GNPs. It is seen that all the GNPs are almost in agglomerated irregular particles, not in the as-expected thin platelets (Figure 1a). In the agglomerates, some GNPs are in a face-to-face closely stacked form (marked by A in Figure 1a,b) and the others are piled up in a disordered form (marked by B in Figure 1a,c). It can be expected that the GNPs agglomerated in form A are quite difficult to disperse due to a larger bonding force between them resulted from large contact area, while those in form B should be relatively easy to be separated because of their small-area contact. In addition, it is also found that there are some compact irregular blocks as shown by Figure 1d, which should be broken graphite particles, i.e., the as-received GNPs include a certain amount of graphite particles that have not been exfoliated into GNPs during production.

**Figure 2:**
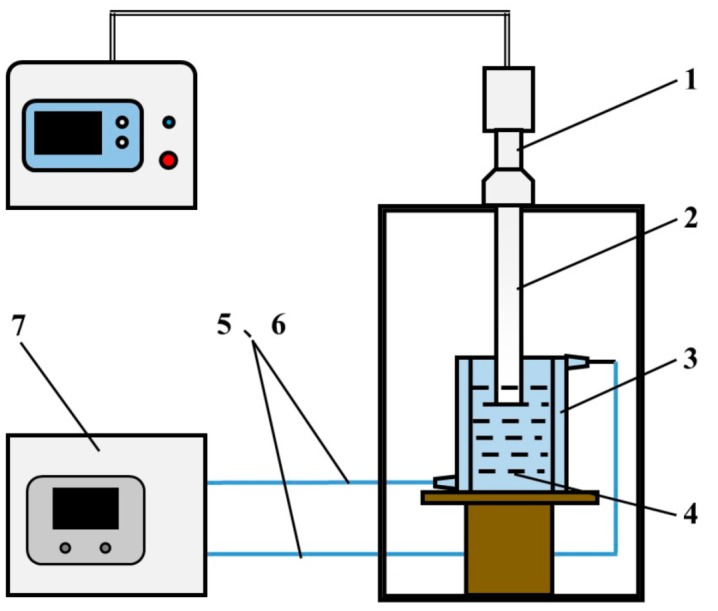
Schematic illustration of ultrasonic treatment. (1—Tip sonicator; 2—Titanium probe; 3—Jacketed beaker; 4—Solvent; 5, 6—Circulating water; 7—Low-temperature thermostatic reaction bath).

A FS-1200N tip sonicator (Shanghai Shengxi Ultrasonic Instrument Co., Ltd., Shanghai, China) was used for the ultrasonic treatment and has power ranging from 0 to 1200 W and a vibrating frequency of 20 kHz, and the amplitude transformer is a titanium bar with a diameter of 20 mm. The used solvent container is a 300 mL jacketed beaker with an internal diameter of 65 mm. The aim for using the jacketed beaker is to control the solvent temperature during ultrasonic treatment by passing circulating water through the jacket. The schematic illustration of the ultrasonic treatment is shown by Figure 2, and the employed treatment parameters are presented in Table 1. The effects of four parameters, ultrasonic time, ultrasonic power, solvent temperature, and solvent kind, were investigated. Three kinds of solvents, ethyl alcohol (EA), isopropyl alcohol (IPA), and tap water, were used to investigate the effects of solvent viscosity and surface tension on dispersion result. The purities of the EA (Tianjin Guangfu Technology Development Co., Ltd., Tianjin, China) and IPA (Tianjin Baishi Chemical Co., Ltd., Tianjin, China) are ≥99.7% and ≥99.7%, respectively. The pH of the tap water is 8.07, and the main contaminants include 229.6 mg/L chloride, 67.2 mg/L sulfate, 0.61 mg/L free chlorine, and 1.61 mg/L nitrate.

**Figure 3:**
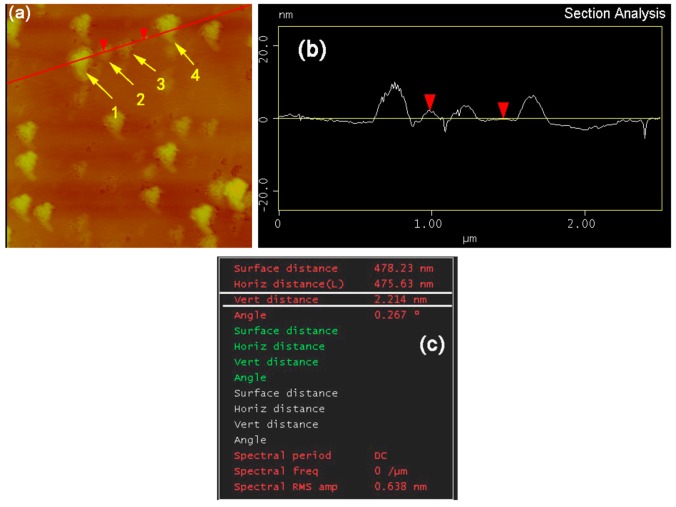
(**a**) AFM image of GNPs, (**b**) examination curve, and (**c**) achieved results.

For the ultrasonic treatment, 200 mg of GNPs and 200 mL solvent were first added into the jacketed beaker, and then the titanium probe was inserted 15 ± 5 mm below the solvent surface in the beaker and treated for a given time at a given power. During ultrasonic treatment, droplets were extracted from the suspension liquid as needed by pipette, and dripped on conductive adhesive tapes, and finally were observed by JSM-6700F SEM, JEOL ltd, Tokyo, Japan) after natural drying. Three typical images with 500×, 1000×, and 2000× magnifications were selected from the edges of each droplet in which the GNPs or agglomerates are individual from each other to quantitatively examine the diameter of GNPs or agglomerates by using Image-pro 6 Plus software (Media Cybernetics Company, Silver Spring, MD, USA). During the examination, each GNP or agglomerate was considered to be a circle that had same area to the GNP or agglomerate. Similarly, to measure the thickness of the GNPs or agglomerates, some droplets were dripped on silicon wafers, and then analyzed by CSPM5500 atomic force microscope (AFM, Being nanometer instrument Co., Ltd., Guangzhou, China) after natural drying. It is noted that some edge regions of each droplet in which the GNPs or agglomerates are individual from each other were selected for AFM analysis as shown by Figure 3a. A line was first drawn on the AFM image (the red line). It is seen that the line encounters four GNPs or agglomerates (marked by 1–4). A curve about the thickness of the four GNPs was then achieved (Figure 3b). Subsequently, the thickness sizes of the four GNPs were examined. For example, as for the thickness of the No. 2 GNP, two points were positioned along the line, one was on the GNP, and the other was on the silicon wafer without GNPs. Then, the thickness value of this GNP, 2.214 nm (i.e., the Vert distance), was obtained as shown by Figure 3c. Repeating the position-fixing process, the thickness sizes of the other three GNPs were examined. The above drawing line, fixing position, and examining datum processes were repeated till the thickness values of all the individual GNPs in this image were examined. The average result of five typical AFM images was taken as the thickness for each droplet.

In addition, some dried droplets were analyzed by Labram HR Evolution Raman spectrometer (HORIBA, Ltd., Tokyo, Japan) to characterize the possible structure damage of GNPs resulting from ultrasonic treatment. The working excitation wavelength was 532 nm at a power of 12 mW, the data were recorded using a grating with 600 lines per mm with a spectral resolution of 0.65 cm^−1^, and the beam spot on the samples was about ~2 μm^2^. To decrease the effect of noise on signal, two cycles of 10 s were performed. The confocal hole was set at about 100 µm and the spectra were collected with a 50× objective. A linear base-line subtraction and intensity normalization were conducted for the resulting spectra. The intensity values of D, G, and 2D peaks, positions of 2D peaks, and full width half maximum (FWHM) were obtained by deconvoluting the data of the Raman spectra using single Lorentz function fit within Origin Pro 9 via the automatic parameter initialization.

An IFS66V/S Fourier transform infrared spectrometer (FT-IR, Brooke technology Co., Ltd., Madison, WI, USA) and a PHI5702 X-ray photoelectron spectrometer (XPS, ULVAC-PHI, Inc. Honshu, Japan) were also used to further clarify the structure change of GNPs occurring during ultrasonic treatment. During the FT-IR analyses, the examinations were carried out in a region of 400–4000 cm^−1^ with a resolution of 4 cm^−1^ on the samples. The resulting spectra were smoothed within Origin Pro 9 using Savitzky–Golay method at 80 points. For the XPS analyses, they were conducted using an aluminum X-ray source (energy 1.4866 keV) and the X-ray spot size was settled at 800 μm with a step size of 0.125 eV.

## 3. Results and Discussion

### 3.1. Effect of Ultrasonic Time

**Figure 4:**
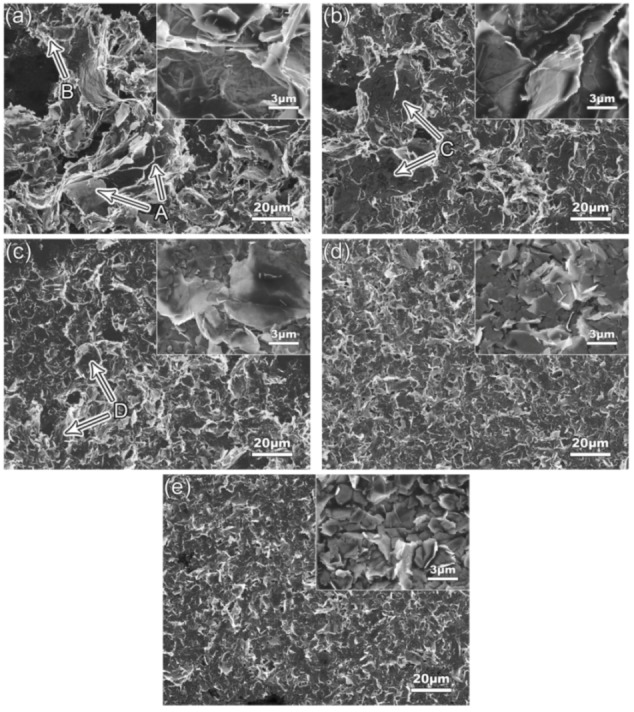
SEM images of GNPs ultrasonically dispersed for (**a**) 1 h, (**b**)2 h, (**c**) 3 h, (**d**) 4 h, (**e**) 5 h. Inserts are the corresponding high-magnification images.

Figure 4 shows the SEM images of GNPs ultrasonic treated at a power of 960 W for different times in EA solvent. It can be seen that compared with the as-received GNPs, the size of the GNPs agglomerates, especially the size and amount of the closely stacked particles (marked by A in Figure 4a), are obviously decreased while the amount of the disordered piled GNPs (marked by B) is vastly increased (comparing Figure 1a and Figure 4a). As the ultrasonic time increases, the size and amount of agglomerates (marked by C and D in Figure 4b,c) continuously decrease (comparing Figure 4a–d). Agglomerated particles disappear and all the GNPs are in individual small-sized platelets when the time reaches 4 h (Figure 4d). As the time is further prolonged, the general morphology almost does not change (comparing Figure 4d,e), but the high-magnification image shows that their diameter size still continues to decrease and their distribution becomes more disordered (comparing the inserts in Figure 4d,e). This means that the further ultrasonic treatment over 4 h possibly brings about the fragmentation of GNPs besides the further improvement of their dispersion. These results indicate that the agglomerated GNPs are gradually dispersed under the actions of shockwaves and shear force generated by ultrasonic cavitation as the ultrasonic treatment proceeds [28], and the fragmentation also simultaneously operates. In addition, it is also found that both the number and size of the large-sized graphite blocks as shown by Figure 1d also decrease and they almost disappear after being treated for 4 h. This indicates that exfoliation of the graphite particles might also occur during treatment and they gradually evolve into GNPs.

**Figure 5:**
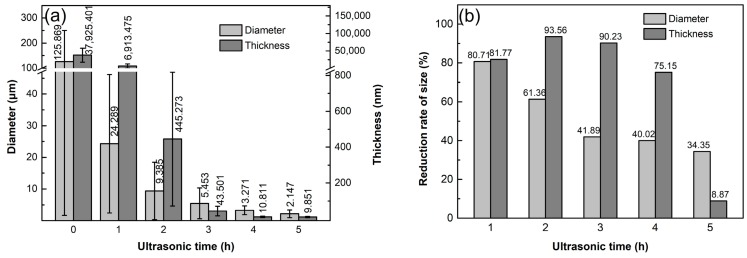
(**a**) Diameter and thickness dimensions, and (**b**) reduction rates of these two parameters of GNPs ultrasonically treated for different times at a power of 960 W.

The change tendencies of the GNPs sizes described above with the ultrasonic time can be more clearly seen from the quantitative examinations shown in Figure 5a. Both the thickness and diameter sizes decrease significantly as the treatment time increases to 4 h, indicating that the average thickness and diameter sizes are decreased from original 125.87 μm and 37925.40 nm to 3.27 μm and 10.81 nm, respectively, which are close to the nominal sizes of 3–10 nm in thickness and 1–3 μm in diameter marked by the manufacturer when the GNPs had just been produced. It is expected that fragmentation, especially exfoliation, can all decrease the thickness and diameter sizes simultaneously besides the dispersion. However, the bonding force between GNPs in the agglomerates should be smaller than that between basal planes in graphite, and the boding between C-atoms in the base planes of GNPs is very strong [1]. Therefore, dispersion should occur more easily than exfoliation, and fragmentation is more difficult to conduct, i.e., the main phenomenon occurred during the initial stage of the sonication should be dispersion. However, the inset high-magnification images in Figure 4b and c show that the diameter of one whole GNP is quite large, while that in Figure 4d indicates that the diameter is very small, in view of the morphologies shown by these images. Therefore, it is suggested that fragmentation might also occur during the ultrasonic treatment, especially after 3 h. In addition, as discussed above, exfoliation might also occur simultaneously. Figure 5b shows that the decrement of thickness at 1 h is about 82%, lower than those of 94% at 2 h and 90% at 3 h. This demonstrates that the main phenomenon occurring during the initial stage should be dispersion, and the exfoliation is enhanced after 1 h. As the time is further prolonged, these two size parameters still continuously decrease, but the decrements are reduced. This can be clearly seen by Figure 5b, the decrement of diameter size is gradually decreased from 80% at 1 h to 34% at 5 h, while that of the thickness is decreased from initial ~90% to 75% at 4 h, and then is significantly reduced to about 9% at 5 h. This means that both the dispersion and exfoliation have basically finished after being treated for 4h, but the fragmentation is still obvious. In addition, Figure 5 also shows that the deviation ranges of these two sizes of the as-received GNPs are very large, and they gradually decrease as the ultrasonic treatment proceeds. That means that the size difference of the agglomerates is very large for the as-received GNPs, and their size gradually becomes increasingly uniform as the sonication dispersion operates. This can be indirectly seen comparing Figure 1a and Figure 4e.

**Figure 6:**
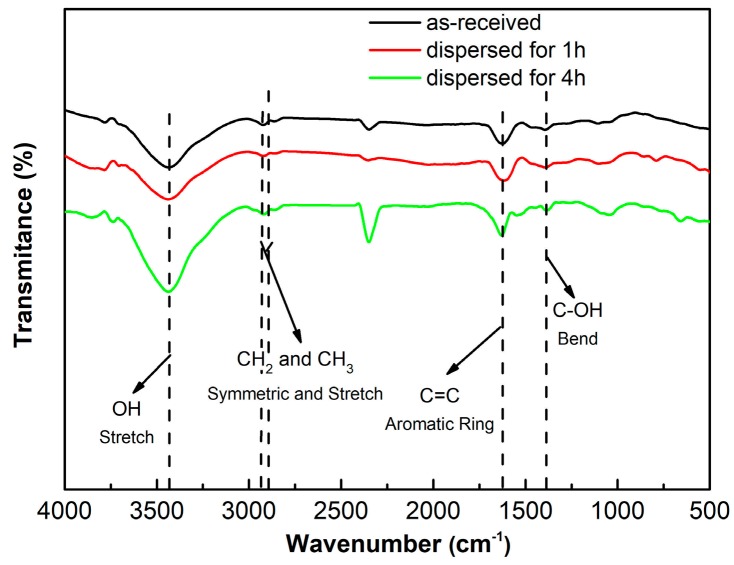
FT-IR results of the as-received GNPs and the GNPs ultrasonically treated for 1 h and 4 h.

To verify the structure change of GNPs from possible chemical reactions occurring during ultrasonic treatment, three typical specimens were analyzed by FT-IR. The result indicates that the change tendencies or the morphologies of the three curves with wavenumber are almost identical, and there is no new band to appear for the two ultrasonically treated specimens compared with that of the as-received GNPs specimen (Figure 6). It is must be noted that for the as-received GNPs, some original GNP powders were directly analyzed by FT-IR, and did not experience the process of dispersion in EA solvent. This implies that no chemical reactions occur between the GNPs and EA during the sonication dispersion. All of the three specimens include four characteristic bands at 2922 cm^−1^, 2852 cm^−1^, 1620 cm^−1^ and 1380 cm^−1^, which correspond to the functional groups of –CH_3_, –CH_2_, C=C and –OH, respectively [30], and they should be generated during production of the GNPs.

**Figure 7:**
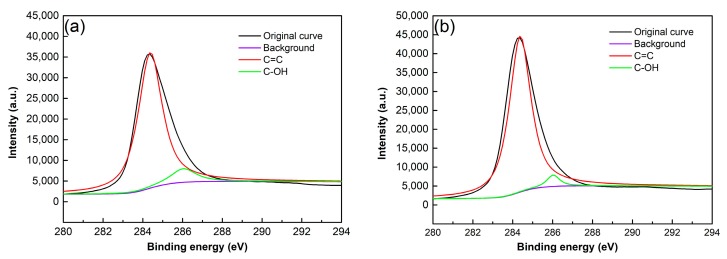
C1s spectra of (**a**) the as-received GNPs and (**b**) the GNPs after 4 h sonication dispersion.

The above analysis from FT-IR only qualitatively suggests that there is no change for the GNP structure due to chemical reaction during the ultrasonic treatment. However, XPS analysis can more affirmatively verify if the structure is destroyed according to the change of the GNP oxygen functional groups content. Figure 7 shows C 1s spectra of the as-received and ultrasonically treated for 4 h GNPs. They all have two characteristic peaks, one at 284.36 eV is attributed to C=C skeleton, and the other at 286.03 eV is resulted from oxygen functionalities [31,32]. Based on the results from the above FT-IR, the oxygen functionality should be hydroxyl (–OH). As shown by Table 2, the amount of C–O in the as-received GNPs is 5.68%, while that in the GNPs ultrasonically treated for 4 h is 4.15%. This means that the hydroxyl content has not obviously changed after being ultrasonically treated. This also indicates that EA solvent does not react with GNPs during the ultrasonic treatment.

**Figure 8:**
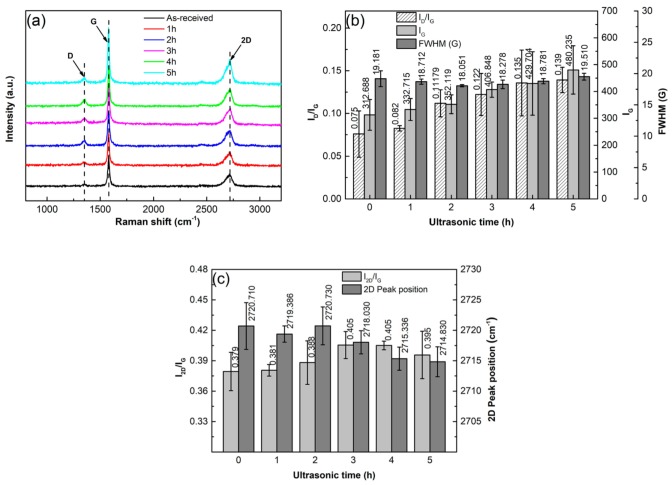
(**a**) Raman spectra of GNPs ultrasonically treated in EA for different times at a power of 960 W, (**b**) quantitative data of I_D_/I_G_, I_G_ and FWHM, and (**c**) quantitative data of I_2D_/I_G_ and 2D peak position.

It is known that Raman spectrum analysis is one of the most commonly used experimental methods to verify and quantify the structural defects of graphene during a processing, such as the formation of bulk defects or topological defects, and fragmentation and exfoliation of GNPs [33,34]. Therefore, the above specimens were also analyzed by Raman spectrometry to further verify if the GNP structure is destroyed during the sonication dispersion. The result shows that each Raman spectrum has three peaks, named G, D, and 2D peaks (Figure 8a). The G peak at ~1580 cm^−1^ results from the vibration of E_2g_ phonons at the center of Brillouin zones and it reflects the crystallinity of graphene, and the higher-intensity I_G_ implies higher crystallinity and lower defect amount [35,36]. The D peak at ∼1350 cm^−1^ indicates in-plane A_1g_ with symmetric breathing modes of rings and its activation requires a defect, so it also can reflect the defect content in carbonaceous materials. The 2D peak at ∼2720 cm^−1^ is the second order of D peak, and its intensity (I_D_) and the ratio of I_2D_/I_G_ are sensitive to the number of graphene layers [37,38]. So the degree of exfoliation can be estimated by the changes in intensity and position of 2D peak and I_2D_/I_G_, the increase of I_2D_/I_G_ and the decrease of 2D peak position indicate exfoliation to occur [39]. According to the implications of D and G peaks, the ratio of I_D_/I_G_, named defect ratio, is always used to characterize the defect amount of carbonaceous materials, the larger the I_D_/I_G_, the higher the defect amount. In addition, the FWHM of G band can also reflect the defect status of graphene in terms of disorder degree of C-atom distribution; the larger the FWHM, the higher the disorder degree of C-atom distribution [40].

To obviously show the change of GNP structure during ultrasonic treatment, the results of I_D_/I_G_, I_G_, and FWHM are quantitatively presented in Figure 8b. It can be seen that both the I_D_/I_G_ and I_G_ continuously increase as the sonication time is prolonged. The increase of I_D_/I_G_ indicates that the defect amount in the GNPs increases during the ultrasonic treatment and the increase of I_G_ means that the crystallinity of the GNPs is improved. The FWHM slightly decreases before 2 h, and then increases slowly, but the values before 4 h are all smaller than that of the as-received GNPs. This indicates that the order degree of C-atom distribution is improved before 2 h, and then the disorder degree is enhanced after 2 h, but the order degree before 4h is still better than that of the as-received GNPs. According to the above discussion, fragmentation of GNPs might occur during the ultrasonic treatment. As expected, the fragmentation should preferentially conduct along the positions with defects such as vacancies or gaps at edges of GNPs. For the former case, the positions with vacancies then become into edges of the fragmented GNPs, and thus, the vacancies, a kind of bulk effects, are decreased. Therefore, the crystallinity or order degree of C-atom distribution is improved, and thus I_G_ is increased and FWHM is decreased. For the latter case, the edges of GNPs are increased, i.e., the defect, the number of C-atoms with dangling bonds, is increased, which results in the increase of I_D_/I_G_. Previous investigations indicated that perfect zig-zag edges could not intensify D peak, but armchair-like edges could do this [40,41]. Therefore, it can be deduced that the increase of I_D_/I_G_ contributes to the increase of armchair-like edges resulted from fragmentation. The continuous increases of I_D_/I_G_ and I_G_ after 2 h imply that fragmentation is still continued after that, which is consistent to the result from the size changes discussed above, resulting in the further increase of armchair-like edges and the continuous improvement of crystallinity, respectively. The above results from FT-IR and XPS analysis show that no chemical reactions occur during the whole sonication process. In addition, there are also no new vacancies because the crystallinity of GNPs is continuously improved. Therefore, it can be suggested that the increase of FWHM after 2 h should only be attributed to the impaired order of C-atom distribution from long-time treatment. The increased disorder degree of C-atom distribution may enhance their chemical activity, and then accelerate the reaction with matrix-alloying elements [42]. Figure 8b also shows that the increment of I_D_/I_G_ is relatively large after 4 h, which further implies that the fragmentation is quite obvious after 4 h.

To verify whether exfoliation occurs in the ultrasonic treatment process, the results of I_2D_/I_G_ and the 2D peak position are quantitatively presented in Figure 8c. Compared with as-received GNPs, it can be seen the ratio of I_2D_/_G_ increases with prolonging the ultrasonic time, and the 2D peak position decreases from 2720 cm^−1^ to 2714 cm^−1^ along with the time. All of these indicate that exfoliation is always operated during the whole sonication process. However, the decrement of 2D peak position after 4 h is very slight, and this further demonstrates that exfoliation after this time is quite weak. Compared with fragmentation, exfoliation is more difficult.

Therefore, it can be concluded that the original large-sized GNP agglomerates are gradually dispersed from each other and separated into uniform GNPs after being ultrasonically treated for 4 h in EA solvent at 960 W. Simultaneously, fragmentation and exfoliation are accompanied, but they are quite weak during the initial 1 h. Fragmentation is still obvious after 4 h although it is significantly weakened as sonication proceeds, while the behavior of exfoliation almost disappears after this time. To keep the structure integrity of GNPs, i.e., to avoid fragmentation, the sonication time should be as short as possible. The fragmentation can enhance the crystallinity of the resulting GNPs but increases the defect of armchair-like edges as well as decreasing their diameter size. The exfoliation leads the graphite left in the original GNPs to evolve into GNPs. There are no chemical reactions to occur and no new vacancies to generate during the sonication process, but long-time sonication treatment can enhance the disorder degree of C-atom distribution.

### 3.2. Effect of Ultrasonic Power

**Figure 9:**
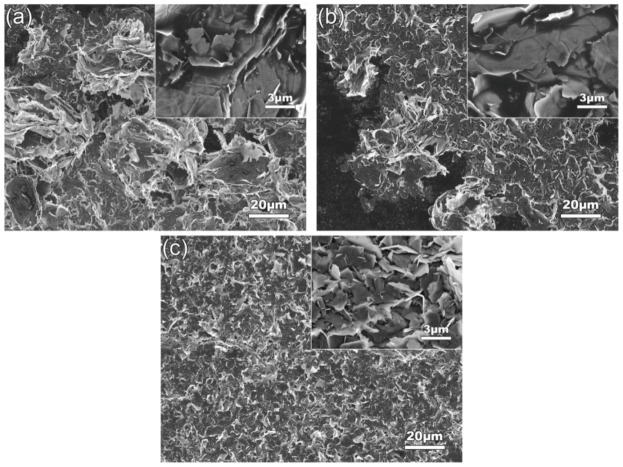
SEM images of GNPs ultrasonically dispersed at (**a**) 720 W, (**b**) 840 W, (**c**) 1080 W. Inserts are the corresponding high-magnification images.

**Figure 10:**
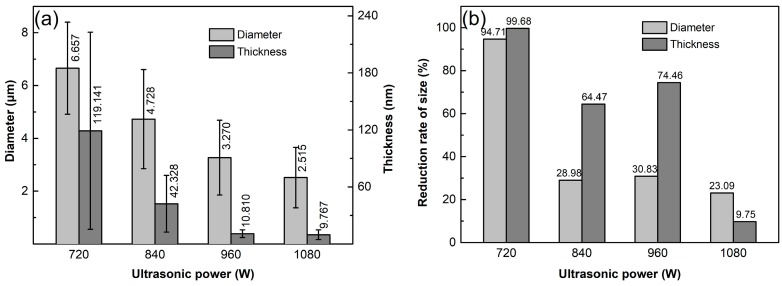
(**a**) Diameter and thickness dimensions of GNPs ultrasonically dispersed for 4 h at different ultrasonic powers, and (**b**) corresponding reduction rates of diameter and thickness.

Figure 9 shows the SEM images of the GNPs ultrasonically dispersed at different powers for 4 h in EA solvent. Together with Figure 4d, it can be seen that similar to the effect of sonication time discussed above, the size of GNP agglomerates gradually decreases with the increase of ultrasonic power, and the agglomerates almost disappear and all evolves into uniform GNPs as the power increases to 960 W (comparing Figure 4d and Figure 9a,c). When the power is further increased, the size of the disordered GNPs is further decreased (comparing Figure 4d and Figure 9c). It is known that the increase of ultrasonic power enhances the amplitude of mechanical vibration, and thus the cavitation effect, resulting in more intensive shockwaves and higher shear force, and so a better dispersion effect and more obvious fragmentation. This change trend can be seen more clearly from the quantitative examinations shown in Figure 10a. The average diameter and thickness dimensions of GNPs decrease steadily with the increase of ultrasonic power. When the power increases from 960 W to 1080 W, the diameter of GNPs decreases from 3.27 μm to 2.52 μm, but the thickness only decreases slightly (from 10.81 μm to 9.76 nm). It is expected that cavitation bubbles from ultrasonic vibration grow too large when the power increases to a certain level, and they do not collapse in time to generate intensive shockwaves and high shear force. In addition, the large-size bubbles can also hinder the transmission of shockwaves, and then the cavitation intensity far away from the vibrating source is weakened [43]. Therefore, the degrees of both fragmentation and exfoliation are decreased. In addition, fragmentation and exfoliation should also become increasingly difficult as the sizes including diameter and thickness of GNPs decrease. Therefore, the resulting fragmentation and exfoliation effects are weakened. This can be demonstrated by the decrements of these two size parameters as shown by Figure 10b: the diameter decrement decreases from about 30% at 840 W and 960 W to 23% at 1080 W, and the thickness decrement decreases from 64% and 74% to 10%. This further demonstrates that fragmentation is occurs more easily than exfoliation. In addition, Figure 10a also shows that the size deviation continuously decreases as the ultrasonic power increases, which means that either the diameter or the thickness of the GNPs becomes increasingly uniform.

**Figure 11:**
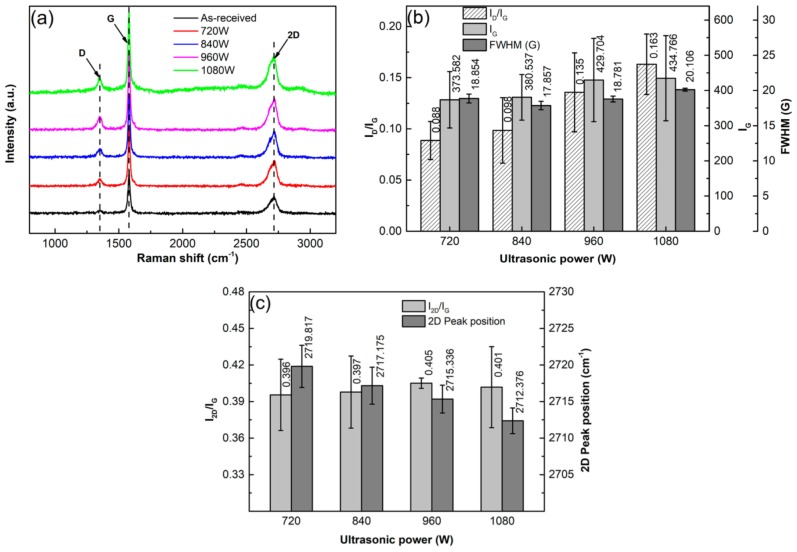
(**a**) Raman spectra of specimens ultrasonically treated for 4 h in EA at different powers, and corresponding (**b**) data of I_D_/I_G_, I_G_, and FWHM, (**c**) data of I_2D_/I_G_ and 2D peak position.

To verify the effect of ultrasonic power on the structure of GNPs, the samples were examined by Raman spectrometry as shown by Figure 11a. The detailed quantitative data of FWHM, I_D_/I_G_, and I_G_ are presented in Figure 11b. It indicates that FWHM slightly decreases as the power increases from 720 W to 840 W and then slightly increases when the power exceeds 840 W. According to the discussion of the above section, it can be suggested that the order degree of C-atom distribution is improved as the power increases from 720 W to 960 W due to the fragmentation of GNPs along the positions with vacancies. It is also just this reason that the crystallinity of the resulting GNPs is enhanced, and thus, the average value of I_G_ slightly increases. Simultaneously, the armchair-like edges are also increased due to the fragmentation, which results in the increase of I_D_/I_G_. However, when power is further increased, the order degree of C-atom distribution is impaired due to the too high-power sonication. The continuous increase of I_G_ implies that the crystallinity is further improved when the power exceeds 960 W. So the increase of defect amount (i.e., the increase of I_D_/I_G_), and the disordering (i.e., the increase of FWHM) are only attributed to the increase of armchair-like edges from further fragmentation and the slight change of C-atom distribution position, and there are no new vacancies to generate even at 1080 W. The data of I_2D_/I_G_ and 2D peak positions are presented in Figure 11c. The general change of I_2D_/I_G_ with increasing the power is in an increasing tendency and the 2D peak position tends to decrease as the power increases, which indicates that increasing the sonication power can accelerate the exfoliation.

In summary, the effects of ultrasonic power on the dispersion effect and structure of GNPs are similar to those of ultrasonic time, i.e., the dispersion effect, including fragmentation and exfoliation effects, are enhanced as the power increases, and simultaneously, the order degree of C-atom distribution is first improved, and then is impaired due to the too high-power sonication. There are no vacancies to form even at 1080 W. It is supposed that the ultrasonic power should be controlled as low as possible under the conditions for guaranteeing the dispersion effect, to avoid fragmentation.

### 3.3. Effect of Solvent Temperature

**Figure 12:**
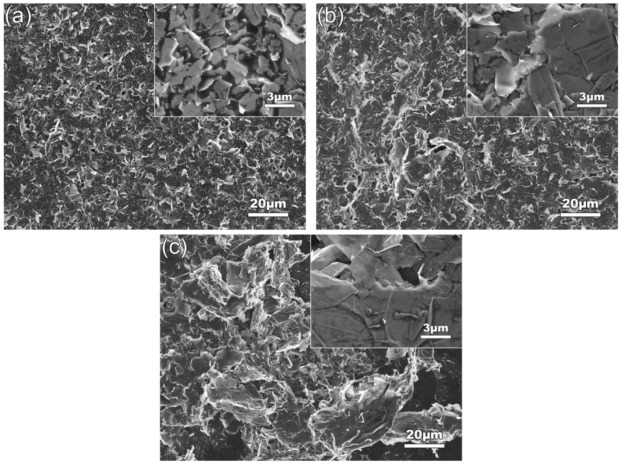
SEM images of GNPs dispersed at (**a**) 25 °C, (**b**) 45 °C, (**c**) 55 °C. Inserts are the corresponding high-magnification images.

**Figure 13:**
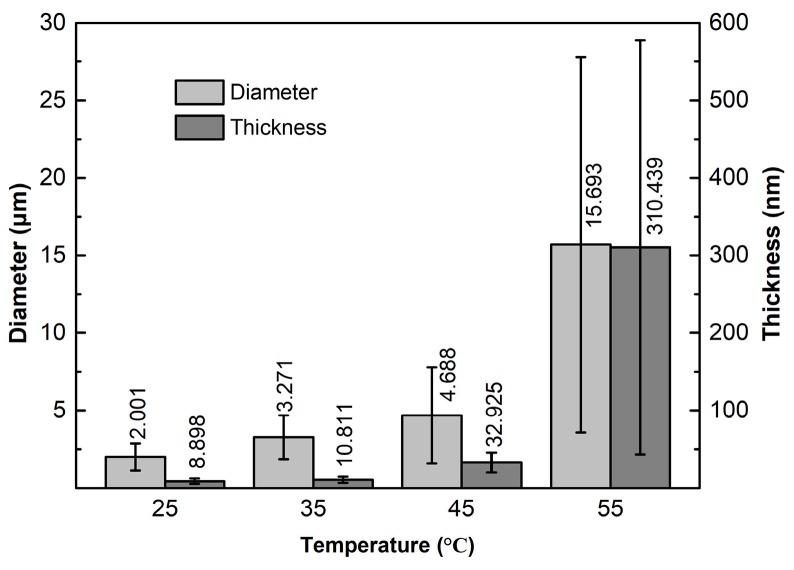
Diameter and thickness sizes of GNPs ultrasonically treated in different temperature EA solvent for 4 h at a power of 960 W.

Figure 12 presents the SEM images of the GNPs ultrasonically treated for 4 h in different temperature EA solvent at a power of 960 W. Together with Figure 4d, it can be seen that the dispersion effect becomes worse and worse as the temperature rises (comparing Figure 4d and Figure 12a–c). As shown by the quantitative examinations in Figure 13, the dispersion efficiency is quite high at 45 °C, but the sizes of the resulting GNPs (4.69 μm in diameter and 32.93 nm in thickness) are larger than the nominal sizes, which means that the GNPs are still not completely dispersed (Figure 12b). When the temperature rises to 55 °C, the GNP size is very large and there are many agglomerates (Figure 12c), and the dispersion effect is very poor. When the temperature reaches 64 °C, the sound made by the ultrasonic treatment becomes very small. In addition, it is found - more and more liquid drops are coagulated on the vessel wall, which implies that a large amount of EA volatilizes at this temperature. The morphologies of the resulting GNPs are basically the same as that of the as-received ones, i.e., there is almost no dispersion effect at this temperature. It is expected that the temperature rise increases the amount of gaseous EA in cavitation bubbles. The cavitation bubbles with gaseous EA not only apply a buffer effect on vibration waves, resulting in the decrease of transfer efficiency of vibration waves, but also do not collapse in time, leading to the decrease of cavitation effect [44], and thus, the dispersion effect is weakened. Therefore, it can be concluded that the low-temperature solvent is beneficial for enhancing the dispersion effect during ultrasonic treatment of GNPs.

**Figure 14:**
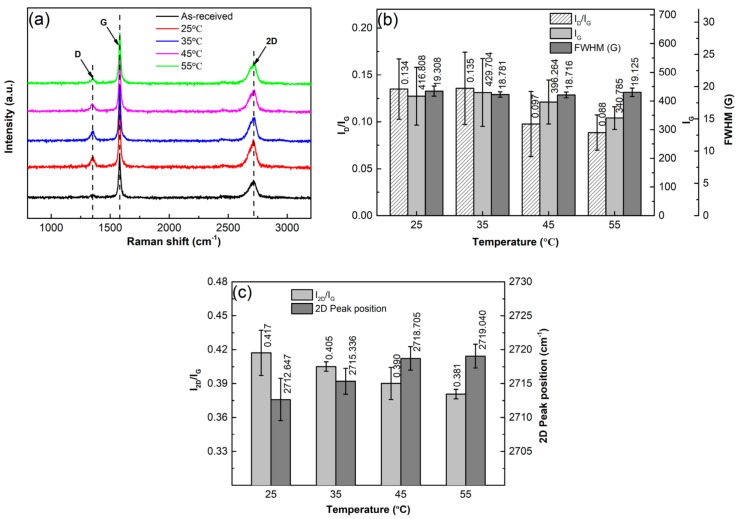
(**a**) Raman spectra of GNPs ultrasonically treated for 4 h in EA at different temperature, and corresponding (**b**) data of I_D_/I_G_, I_G_, FWHM, and (**c**) data of I_2D_/I_G_, and 2D peak position.

To clarify the effect of solvent temperature on the structure of GNPs, the samples were also examined by Raman spectrometer as shown by Figure 14. It is found that the value of FWHM slightly decreases as the temperature rises from 25 °C to 45 °C, and then slightly increases (Figure 14b). As discussed above, the higher the temperature, the poorer the dispersion effect, i.e., the smaller the force acted on GNPs. Therefore, at the lowest temperature of 25 °C, the force acted on GNPs is the largest, and thus, the effect on C-atom distribution is the largest, which results in the increase of disorder degree of C-atom distribution, and thus, the relatively large value of FWHM. As the temperature rises, the force acted on GNPs is gradually decreased, and then the order degree of C-atom distribution is improved, so the value of FWHM is increased. However, when the temperature exceeds 45 °C, the fragmentation of GNPs becomes quite weak due to the decreased force, and the status of the resulting GNPs is close to that of the original ones. From the above discussion, it is known that the order degree and the crystallinity of the original GNP are not very high. So FWHM slightly decreases again when the temperature exceeds 45 °C. It is just due to the decreased fragmentation that the generated armchair-like edges are decreased, and the decrement of some defects, such as the vacancies that exist in the GNPs, are reduced, i.e., the crystallinity is decreased, so both I_D_/I_G_ and IG decrease with the rise of temperature (Figure 14b). The quantitative results of I_2D_/I_G_ and the 2D peak position are presented by Figure 14c. It shows that I_2D_/I_G_ continuously decrease while 2D peak position continuously increases as the temperature rises. All of these imply that the exfoliation degree is weakened with rising the temperature. As discussed above, this should be attributed to the decreased force enacted on the GNPs from the temperature rise, i.e., the results from Raman spectrometry demonstrate that rising solvent temperature also decreases the degrees of fragmentation and exfoliation besides the dispersion effect.

In summary, the higher the solvent temperature, the lower the dispersion efficiency, also including the fragmentation and exfoliation of GNPs, and these three affects almost completely disappear when the temperature of EA solvent reaches 55 °C. To achieve a good dispersion effect, the temperature cannot exceed 35 °C.

### 3.4. Effect of Solvent.

**Figure 15:**
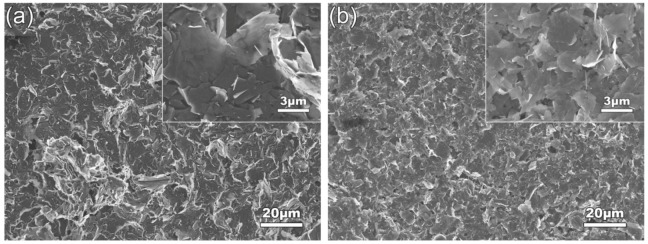
SEM images of GNPs dispersed in (**a**) IPA solvent, (**b**) Tap water solvent. Inserts are the corresponding high-magnification images.

**Figure 16:**
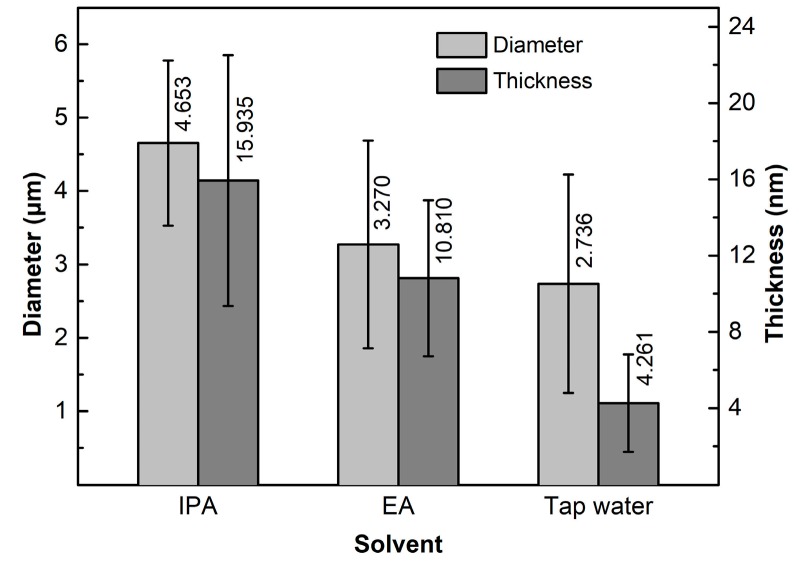
Diameter and thickness dimensions of GNPs ultrasonically dispersed in different solvents for 4 h at a power of 960 W.

To clarify the effects of solvent viscosity and surface tension on the dispersion of GNPs, the other two solvents of IPA and tap water were also used. Table 3 givens the viscosities and surface tensions of the three solvents at 20 °C [45,46]. It shows that the viscosity of IPA (2.43 mPa·s) is obviously larger than that of EA (1.17 mPa·s), and their surface tensions are almost same (21.70–22.27 mN/m), while the surface tension of tap water (72.67 mN/m) is significantly higher than that of EA (22.27 mN/m), and their viscosities are basically same (1.01–1.17 mPa·s). Therefore, the effect of solvent viscosity on dispersing GNPs can be obtained by comparing the statuses of GNPs in EA and IPA solvents, and the effect of surface tension can be achieved by comparing those in tap water and EA solvents. Figure 15a shows the SEM images of GNPs dispersed in IPA solvent at 960 W for 4 h (EA solvent shown in Figure 4d). Compared to the image in EA solvent as shown by Figure 4d, it can be seen that the dispersion effect in EA is obviously better than that in IPA, which indicates that low-viscosity solvent is beneficial for dispersion of GNPs. The quantitative examinations presented in Figure 16 indicate that both the diameter and thickness sizes of GNPs in EA solvent are all smaller than those in IPA solvent. It is known that the hindering role of a solvent to transfer of vibration waves increases with increasing solvent viscosity, and the formation of cavitation bubbles and their collapse in time are also lagged with the viscosity, and thus, the dispersion effects, including fragmentation and exfoliation effects, are decreased [47]. Figure 15b gives the SEM micrograph of the GNPs treated in tap water. It is seen that the morphologies of GNPs are very similar to those in EA solvent (comparing Figure 4d and Figure 15b). However, Figure 16 indicates that the dispersion effect in tap water is obviously better than that in EA, the diameters, especially the thickness in the former solvent, are distinctly smaller than those in later ones. During the experiments, it is found that the color of the resulting GNP suspension liquid in tap water is obviously lighter than that in EA. It is known that the light transmittance of GNPs is improved as the layers in GNPs decrease, and the light transmittance of single-layer graphene is as high as 97.7% [48]. This phenomenon means that the thickness of GNPs treated in water is thinner than that in EA, which also indicates that the dispersion effects, including the exfoliation effect in tap water, are better than those in EA. With the increase of liquid surface tension, the shrinkage force of cavitation bubbles increases, i.e., cavitation strength is improved, and thus, the sonication effect is enhanced [49]. In addition, the GNPs used in this work have functional group of –OH as discussed above, which makes the GNPs hydrophilic [32]. Therefore, tap water is easier to infiltrate into inter-GNPs in agglomerates in order to separate them from each other, i.e., tap water has a better dispersion effect, especially an exfoliation effect, i.e., a solvent with high surface tension is conducive to improving ultrasonic dispersion efficiency, and tap water—one of the most common solvents with a cheap price—is an ideal solvent for dispersion of GNPs only in view of dispersion and exfoliation effects. However, the shortcomings of tap water are also obvious: it is not easy to dry compared with EA, and also can react with some active metals such as Mg alloys during fabrication of their composites [50], i.e., tap water is not suitable for dispersing GNPs during fabrication of active metal-based composites.

**Figure 17:**
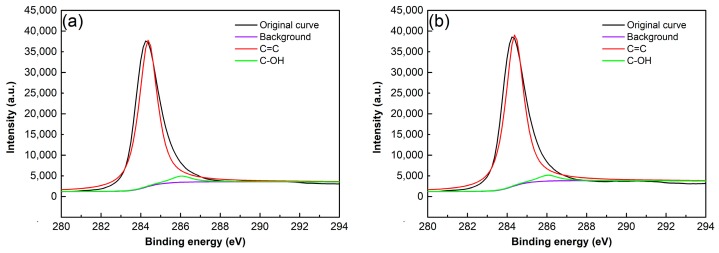
C1s spectra of GNPs dispersed in (**a**) tap water solvent, and (**b**) IPA solvent.

Figure 17 presents the C1s spectra of the GNPs treated in tap water and IPA solvent, which shows that they also have two characteristic peaks of C–C skeleton and C–O bond. The contents of C–O bond in the GNPs treated in these two solvents are basically equivalent to that of the as-received GNPs (comparing Table 2 and Table 4), i.e., the content of –OH group almost does not change after being ultrasonically treated in these two solvents. Therefore, it is certain that GNPs also do not react with these two solvents during the ultrasonic treatment.

**Figure 18:**
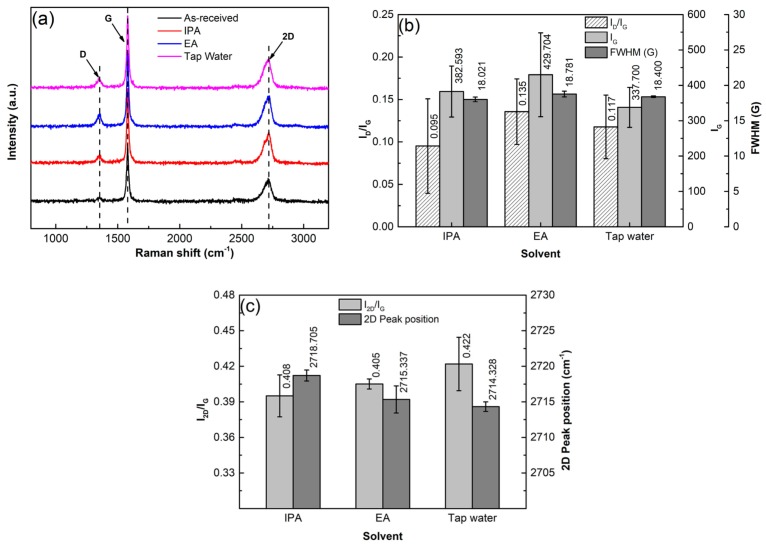
(**a**) Raman spectra of GNPs ultrasonically treated in different solvents for 4 h at a power of 960 W, and corresponding (**b**) data of I_D_/I_G_, I_G_, FWHM, (**c**) data of I_2D_/I_G_, 2D peak position.

Raman spectrometry is also used to investigate the effects of surface tension and viscosity on the structure of GNPs as shown by Figure 18a, and the detailed data are presented in Figure 18b,c. It is seen that the data of I_D_/I_G_, I_G_, and FWHM of the GNPs treated in IPA are all smaller than those in EA (Figure 18b), which means that the defect amount, crystallinity, and order degree of C-atom distribution of the former GNPs are all higher than those of the former ones. According to the implications of these parameter changes and their relationships with the behaviors of GNPs during ultrasonic treatment discussed in the above sections, this status should result from the lower degrees of fragmentation and exfoliation in IPA than in EA. This is just consistent with the result achieved from the above discussion about the size variations, i.e., the solvent with lower viscosity (i.e., EA) is beneficial for dispersion, and can enhance fragmentation and exfoliation simultaneously. Figure 18b shows that the three parameters, I_D_/I_G,_ I_G,_ and FWHM of the GNPs treated in tap water are also smaller than those in EA. The result from the above size changes suggests that the used GNPs have a given hydrophilicity, and the functional groups on the surfaces of GNPs can absorb H_2_O molecules, and then a layer of H_2_O molecules may form on their surfaces. On the one hand, these layers separate the GNPs from each other and the dispersion effect is enhanced; on the other hand, they equivalent act as protection layers, which avoid the GNPs suffering from the large impact effect of vibration waves. Therefore, it can be expected that the fragmentation degree and the disorder degree of C-atom distribution are also decreased, and thus, I_D_/I_G_, I_G_, and FWHM are smaller than those in the EA solution. Based on this standpoint, it is suggested the size decreases of GNPs compared with those in EA solvent should mainly be attributed to exfoliation, but not fragmentation. Therefore, it can be concluded that tap water is beneficial for maintaining the structural integrity of GNPs during the sonication process. This is just due to the good exfoliation effect in tap water and that I_2D_/I_G_ and 2D peak position of GNPs are larger and smaller than those in EA solvent, respectively (Figure 18c). However, in IPA solvent, exfoliation effect is smaller than that in EA solvent, so I_2D_/I_G_ and the 2D peak position of GNPs are smaller and larger than those in EA solvent, respectively.

Therefore, it can be concluded that the solvents with lower viscosity or larger surface tension not only can improve dispersion efficiency of GNPs, but can also enhance exfoliation and fragmentation degrees. For tap water, a high-surface-tension solvent, fragmentation is quite weak, but exfoliation is obvious due to the hydrophilic characteristic of GNPs while maintaining a good dispersion effect. However, it does not easily dry, and may react with some active metals (i.e., Mg alloys) during fabrication of these metal matrix composites. Therefore, EA should be the most suitable solvent among the solvents involved in this work.

## 4. Conclusions

(1) Increasing ultrasonic time or power is beneficial for dispersion of GNPs, but can also enhance fragmentation degree as well as improving exfoliation effect. Fragmentation not only results in the formation of a kind of defect of armchair-like edges, but also decreases the aspect ratio of GNPs, although crystallinity of the fragmentated GNPs can be slightly improved. In addition, too long-term or large-power sonication can increase the disorder degree of C-atom distribution. Therefore, the time or power should be decreased as much as possible to guarantee the dispersion effect.

(2) Solvents with low temperature, low viscosity, or high surface tension are beneficial for improving dispersion and exfoliation of GNPs, but simultaneously enhance fragmentation. For tap water, a high-surface-tension solvent, it has relatively low fragmentation degree and good dispersion and exfoliation effects due to the hydrophilicity of GNPs. However, it has obvious shortcomings, such as difficulty in drying and possible reaction with some active metals.

(3) There are no new vacancies to form and no chemical reactions to occur during ultrasonic treatment in EA solvent. In view of the dispersion effect and volatility, EA is the best solvent among the three solvents of EA, IPA, and tap water. GNPs can reach the expected status when they are ultrasonically treated for 4 h under a power of 960 W in EA solvent at 35 °C.

## Figures and Tables

**Table 1:** Ultrasonic treatment parameters used in this work.

Effect Factor	Ultrasonic Time (h)	Ultrasonic Power (W)	Solvent Temperature (°C)	Solvent
Ultrasonic time	1;2;3;4;5	960	35	EA
Ultrasonic power	4	720; 840; 960; 1080	35	EA
Solvent temperature	4	960	25;35;45;55	EA
Solvent kind	4	960	35	EA; IPA;Tap water

**Table 2:** The amounts of C=C and C–O groups in GNPs.

Specimen	Amount (%)	
	C=C	C–O
As-received GNPs	94.32	5.68
Ultrasonically treated for 4 h	95.85	4.15

**Table 3:** Viscous coefficient and surface tension of the different solvents.

Solvent	Viscosity (mPa·s)	Surface Tension (mN/m)
EA	1.17	22.27
IPA	2.43	21.70
Tap water	1.01	72.67

**Table 4:** The amounts of C–C and C–O groups in the GNPs treated in tap water and IPA solvent.

Specimen	Amount (%)	
	C–C	C–O
Treated in tap water	95.41	4.59
Treated in IPA solvent	95.86	4.14
